# Chemical Remarks on Manures

**Published:** 1799-03

**Authors:** S. L. Mitchill

**Affiliations:** New York


### *Chemical Remarks on Manures*:—*By Dr.* S. L. Mitchill, *of New York*.

IN an ingenious and elaborate eſſay, publiſhed in the "*Medical Repoſitory*," of New York, the author has endeavoured to prove, how nearly *phyſic* and *farming* are allied to each other. He premiſed an inquiry into the nature of *ſepton* (azote), and its relation to other bodies; and after having ſtated the opinions entertained in different ages with reſpect to this acid, which at one time was termed *acidum primogenium*, at another *acidum univerſale, acidum vagum, acidum atmoſphericum, acidum æreum, &c. &c.* Dr. Mitchill directs his inquiry into the particular phœnomena, which a country rendered unhealthy by its uncommon fertility, or a dung-heap, on account of its richneſs, preſents to the mind; and, in order to illuſtrate the hiſtory of azote, or the principle of putrefaction in manures, he gives a liſt of forms or combinations, in one or the other of which this principle exiſts, either in a pure or combined ſtate. Our limits not permitting us to tranſcribe the particulars particulars of this interefting difquifition, in which the Doctor enumerates the principal compofitions of *fepton* with other bodies which relate to agriculture, and accompanies each of them with remarks tending to illuftrate their refpective value, as well as their relation to other fubftances, we fhall extract only the following:

" The acid properties of fome manures, and the alkaline or abforbent nature of others, highly deferve the confideration of agriculturifts. Without paying attention to the action of the former, of thefe upon the latter, the formation of manures, and their operation upon plants, cannot be underftood. Thefe might eafily be reduced to a fmall table, and their affinities traced.

| *Acids commonly prefent in Manures.* | *Alkalies very common in Manures.* |
|---|---|
| *Septic*, or nitrous acid. | *Pot-Afh*, or *vegetable* fixed alkili, very common. |
| *Carbonic* acid, or fixed air, both very common. | *Soda*, or mineral alkali. |
| *Sulphuric* acid, fometimes. | *Ammoniac*, or volatile alkali, now and then. |
| *Muriatic* acid, very frequent in the neighbourhood of the ocean, and abounding on farms where much falt-fodder is confumed. | *Lime*, or calcareous earth, very common indeed. |
| *Phofphoric* acid, not unfrequently evolved during animal decompofition. | *Magnefia*, fometimes. |
| | *Clay*, almoft always." |

In the fecond number of the American "*Medical Repofitory*," we meet with a letter written by the fame author, and addreffed to N. Webster, Jun. from which we communicate the following remarkable paffages:—" It muft be a welcome intelligence, that the collected mafs of nuifance which we now are, with fuch happy fuccefs, engaged in removing from the city of New York, is convertible, by the powers of vegetation, from poifon to wholefome articles of food. There muft be high fatisfaction in contemplating how the purity and healthinefs of the towns may contribute to the thriftinefs and wealth of the furrounding country. And to all of us it is matter of the utmoft moment, to receive additional proofs of the power of the alkaline qualities of the *lime, pot-afh*, and *foda, thrown out and fcattered about the ftreets, to neutralize the acid vapours which excite fevers and plagues among us**, and convert them into the *richeft of manures;* and thus, by one operation,

* Dr. Hermbstaedt of Berlin has lately given additional confirmation to the extensive utility of this simple process of purifying the atmosphere. He directs his asthmatic and consumptive patients, to expose pot-ash or pearl-ash in a *dry* and *powdered*

operation, clearing the atmosphere of its noxious fumes, and preparing nourishment for the vegetable world.

"I hope one day to be able to add to these testimonials, the result of my own experience on these carbonates and septites of lime, pot-ash, and soda, in raising crops of barley and wheat; and in themean time entertain the hope, that further particulars concerning the operation of *street-manure*, in raising Indian corn, will be given us by the gentlemen who have introduced the experiments*. I think we are getting on the right track of inquiry about these matters, and shall soon be able, for it is most certainly in our power, to make pestilence submit to municipal and agricultural regulations."

*dered* state, upon glass or china plates, one inch deep, to the air of bed-chambers and sitting-rooms: in the course of four weeks the pot-ash will be completely saturated with aerial acid, or cabonic acid gas, the most irrespirable part of the atmosphere.

* In one of these experiments we are informed, that the crop raised by Mr. John Stevens of Hoboeken, amounted to 118 bushels and two quarts of indian corn (in the ears) per acre; in another, by Daniel Ludlow, of Westchester, the produce was, upon an average, 98 bushels and 14 quarts per acre;...but much greater was the fecundity of the soil in the neighbourhood of Elizabeth-Town, where 155 bushels of Indian corn in the ears have been produced by *one* acre of land: and this, it is added, falls vastly short of what *may* be obtained from an equal quantity of ground.

